# Adversity, social capital, and mental distress among mothers of small children: A cross-sectional study in three low and middle-income countries

**DOI:** 10.1371/journal.pone.0228435

**Published:** 2020-01-30

**Authors:** Jewel Gausman, S. Bryn Austin, S. V. Subramanian, Ana Langer

**Affiliations:** 1 Department of Social and Behavioral Sciences, Harvard TH Chan School of Public Health, Boston, MA, United States of America; 2 Division of Adolescent and Young Adult Medicine, Department of Social and Behavioral Sciences, Harvard TH Chan School of Public Health, Boston Children’s Hospital, Boston, MA, United States of America; 3 Department of Social and Behavioral Sciences, Harvard TH Chan School of Public Health, Boston, MA, United States of America; 4 Women and Health Initiative, Department of Global Health and Population, Harvard TH Chan School of Public Health, Boston, MA, United States of America; Bielefeld University, GERMANY

## Abstract

**Background:**

Maternal mental health is becoming recognized as a global health priority. Mental distress among mothers of young children may be exacerbated by exposure to adversity. Social capital may buffer the impact of adversity on mental distress during the postnatal period and beyond. This paper examines the relationship between adversity, cognitive social capital and mental distress among mothers of young children in three low and middle-income countries.

**Methods:**

This study uses data from the Young Lives study on 5,485 women from Ethiopia, India, and Vietnam. Logistic regression was used to examine the association between exposure to stressful life events (SLEs) and mental distress in women between 6 months and 1.5 years post-partum. Logistic and linear regression was used to examine the potential for effect modification by social capital.

**Results:**

The proportion of women with mental distress during the period between 6–18 months following the birth of a child in the sample was 32.6% in Ethiopia, 30.5% in India and 21.1% in Vietnam. For each additional SLE to which a woman was exposed, the odds of MMD increased by 1.28 (95% CI: 1.22, 1.36; p<0.001) in Ethiopia, 1.17 (1.11, 1.25; p<0.001) in India, and 1.98 (1.75, 2.25; p<0.001) in Vietnam. Exposure to family SLEs was significantly associated with MMD in all three countries with odds ratios of 1.76 (95% CI: 1.30, 2.38; p<0.001), 1.62 (95% CI: 1.12, 2.33; p<0.01 in India), 1.93 (95% CI: 1.27, 2.92; p<0.01), respectively. In Ethiopia and India, economic SLEs were also significantly associated with MMD after adjustment (Ethiopia OR: 1.68; 95% CI: 1.12, 2.52; p<0.01 and India OR: 1.44; 95% CI: 1.01, 2.05; p<0.05), while in India, crime SLEs (OR: 1.93; 95% CI: 1.27, 2.92; p<0.01) were associated with MMD. Cognitive social capital was found to modify the association between SLEs and symptomology of mental distress in Ethiopia, India and Vietnam.

**Conclusions:**

This study suggests that adversity may increase the risk of maternal mental distress in three LMICs, while social capital may buffer its effect.

## Introduction

Depression, anxiety, and other common mental disorders among mothers during the perinatal period and beyond are becoming increasingly recognized as important global health priorities [1]. The inclusion of mental health in the Sustainable Development Goals speaks to the importance of mental health among women in low and middle income countries (LMICs) [2]. Depression is the second leading cause of disease burden in women globally and manifests in ways that can have a profound impact on maternal functioning and parental role [3]. While data on mental distress during the perinatal period remains limited in LMICs [2], a systematic review of perinatal mental disorders among women in LMICs estimated an average prevalence of 15.6% antenatally and 19.8% postnatally [4]. Mental distress during the antenatal period, post-partum period and beyond can have lifelong consequences for women, their children and their families. Women who suffer from one postpartum episode of major depression have a 25% risk of it reoccurring later in life [5]. Further, mental distress during the perinatal period is associated with myriad biological, cognitive, social consequences related to infant and child development [6].

Maternal mental distress (MMD) in the first years of a child’s life may be related to a combination of biological, social, and environmental factors, including food insecurity, perinatal infections, attributes of living conditions, and violence [7]. Biological change during the perinatal period may result in increased sensitivity to external stress, thereby increasing a woman’s vulnerability to MMD [8]. Stressful life events (SLEs) are events that negatively influence the way a household typically lives [9]. A small body of literature, primarily conducted in high income countries, examines the association between exposure to SLEs and MMD [10–14]. A study in Nepal found that women 5–10 weeks postpartum who experienced a SLE in the year prior to the study had 4.6 times the odds of postnatal depression compared to women who had not experienced a SLE [15]. Another study conducted among 162 women in Nigeria found an association between postnatal emotional disorders and exposure to marital and family-related adverse events [16].

Identifying mechanisms to overcome adversity by fostering resilience has been a focus of recent research on improving mental health outcomes [17]. Social capital may be one mechanism through which the community social environment contributes to the individual coping response [18, 19] to mitigate the effect of adversity. Social capital commonly refers to the ability to secure resources, material or otherwise, through membership in social networks or structures [20]. Cognitive social capital (CSC) refers to an individual’s perceptions of the values, attitudes and beliefs that produce trust, cooperative behavior and norms of reciprocity in their community [21]. CSC may reduce the negative effects of SLEs on mental health by increasing an individual’s sense of belonging, self-esteem, and supportive resources [22]. A previous analysis using data from the Young Lives Study suggests that CSC may reduce the odds of MMD, but it did not examine the effect of adversity on MMD or the potential for effect modification between adversity and CSC on MMD [23]. A few studies have found support for a similar interaction with regard to major depressive disorders [24, 25]; however, this hypothesis has not been examined extensively as it relates to MMD. One study examining the potential for social support to buffer the mental health effect of SLEs among mothers of young children did not find evidence for an interactive effect [19].

The purpose of this paper is to examine the relationship between adversity, cognitive social capital and MMD by using a cross-country comparative perspective focused on three LMICs. Specifically, this paper will 1) examine the association between the number of SLEs experienced and MMD, 2) decompose the association between MMD and the type of SLE suffered, including family, economic, crime, and environmental, and 3) examine the potential for CSC to modify the association between SLEs and MMD.

## Methods

### Study population

The data used in this study are from the younger cohort of the Young Lives Study (YL) that has collected longitudinal data on 2,000 children and their caregivers in each country since 2002 in Ethiopia, India, Peru, and Vietnam, beginning when the index child was aged one year. Ethical approval for this study was obtained through the London School of Hygiene and Tropical Medicine Ethics Committee, and in-country institutional review boards [26]. YL uses a cluster design in which 20 clusters were selected in each country and 100 children were randomly selected from each site [27]. Children were eligible to participate if they were aged between 6 and 18 months. Cluster selection was non-random and based on the cluster’s overall poverty status [28–30]. All women with complete data on all variables were included in this analysis. Data on the outcome variable was not available in Peru.

### Outcome of interest: Maternal mental distress

**Fig 1:**
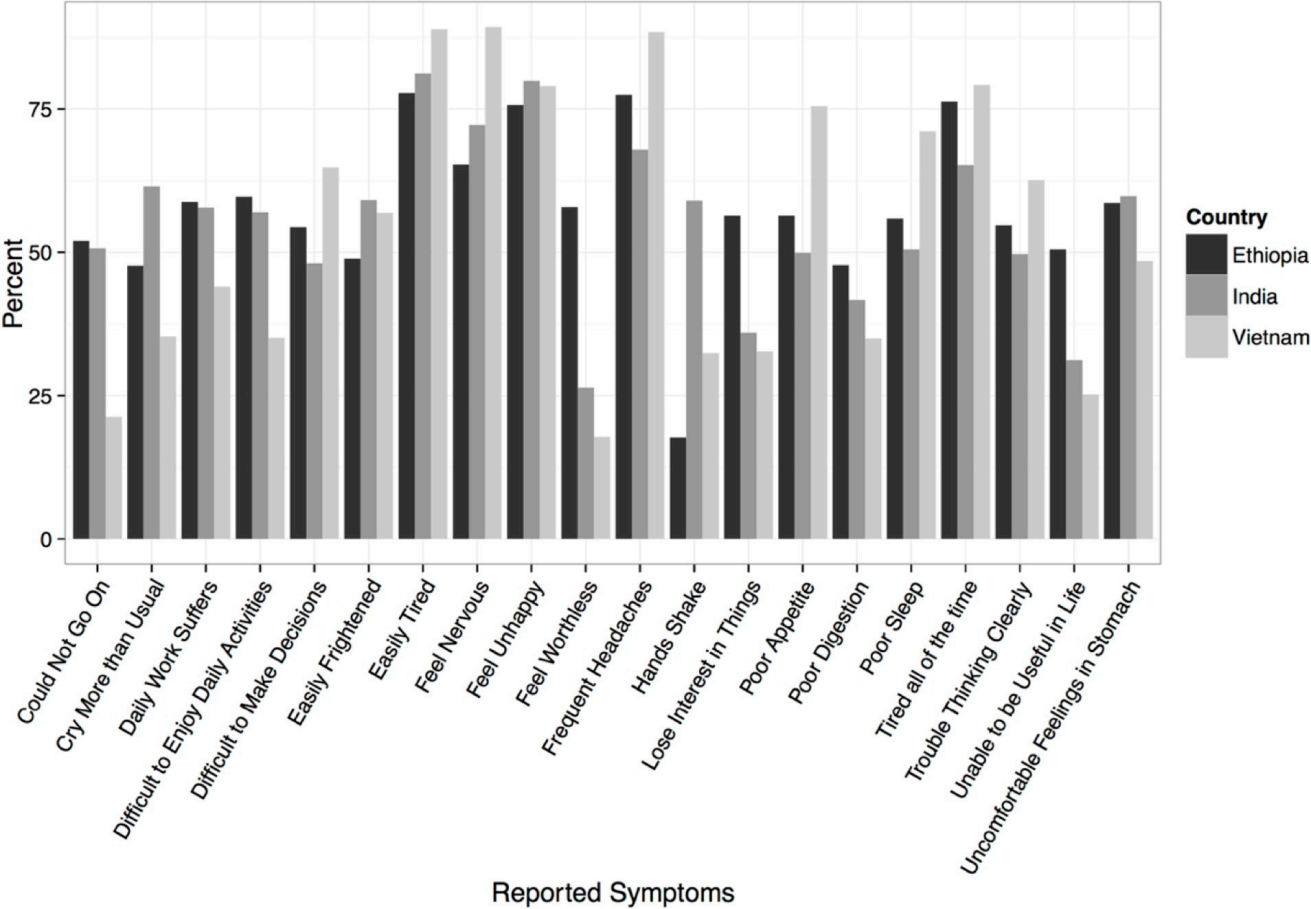
Proportion of respondents reporting specific symptoms of maternal mental distress in Ethiopia, India, and Vietnam as assessed by the Self-Reporting questionnaire-20.

MMD was assessed using the 20-Question Self-Reporting Scale (SRQ-20) which was developed and validated by the World Health Organization [31]. The SRQ-20 was included as part of a larger survey which was administered verbally by a trained interviewer when the mother was between 6 and 18 months post-partum. This study uses previously validated cut-off scores whereby a woman is considered to have mental distress if she answers positively to at least 7 (in India) or 8 (in Ethiopia or Vietnam) of the 20 questions [32–34]. Fig 1 provides a list of the SRQ-20 questions. We examine probable cases of MMD by constructing a binary variable indicating whether a woman’s SRQ-20 score is above or below the pre-defined threshold for her country. In addition, we examine a woman’s SRQ-20 score as a continuous outcome (ranging between 1 and 20 points).

### Exposures of interest: Stressful life events (SLEs) and Cognitive Social Capital (CSC)

The exposure of interest is exposure to SLEs during the prenatal period. Exposure to SLEs was ascertained by a woman’s self-reported experience through recall at 6–18 months post-partum of an economic-related SLE (death of livestock, loss of a job/source of income/family enterprise, or decrease in food availability), an environmental-related SLE (crop failure or a natural disaster), a family-related SLE (divorce or separation, birth of a new household member, enrollment of a child in school, death/reduction of household member(s), severe illness or injury, or move/migration), or crime-related SLE (theft of crops/livestock or being the victim of another crime) in the 12 months before childbirth. A summary, continuous variable was created based on the cumulative total SLE exposure. A binary variable was created for each type of SLE the woman experienced.

CSC was assessed through questions regarding trust, social harmony, perceived fairness, and sense of belonging using the Short Social Capital Assessment Tool (SASCAT), which was specifically designed for use in LMICs and validated in Peru and Vietnam [35]. In this study, CSC was used as a continuous variable ranging from 0–4 points depending on a woman’s response to the four SASCAT questions, with 0 being the lowest level of CSC.

### Other explanatory variables

The other covariates in this study include standard demographic variables that may relate to the experience of both stressful life events and MMD, such as residence (rural/urban), maternal age, parity, marital/cohabitation status (living with partner, married but living apart from partner, single, or divorced/separated/widowed), maternal education (completed primary school or not), time living in the community, member of religious/ethnic majority, household composition (number of children under 5 years, number children between 5–15 years, and number of individuals older than the age of 16 years), and whether the mother is the head of household as they may confound the relationship of interest. Wealth quintile was constructed as a composite, asset-based index [36].

### Statistical procedures

Descriptive statistics were calculated to explore the distribution of relevant characteristics among women included in the study. We first used logistic regression analysis to examine the unadjusted associations between the number of SLEs experienced and the presence of MMD. Second, we used backward elimination, with a p-value threshold of 0.2, to determine which covariates to retain in the final multivariable model (Eq 1) [37]. Backward elimination was conducted separately in each country. We decided a-priori to retain wealth quintile and maternal age given their theoretical importance. The general logistic regression equation is presented for Model 1, in which *X*_2_…*X_n_* represent the covariates included in the model.

$$\log(\frac{MMD}{1-MMD})=\beta_{0}+\beta_{1}(Number\;of\;SLEs)+\beta_{2}X_{2}\ldots \beta_{n}X_{n}+e$$ (1)

Second, we examined the association between experiencing different types of SLEs (environmental, economic, family, and crime events) and MMD. We first examined the unadjusted associations between MMD and exposure to each type of SLE using simple logistic regression. We then extended Model 1 to include the binary variable as to whether a woman experienced a specific type of SLE (Eq 2) in which *X*_3_…*X_n_* represent the covariates included in the model. Last, in order to adjust for the possibility that SLEs may occur in tandem with one another, we simultaneously adjust for the co-occurrence of multiple types of SLEs by including them each in the model (Eq 3), whereby *X*_6_…*X_n_* represent the covariates included in the model.

$$\log(\frac{MMD}{1-MMD})=\beta_{0}+\beta_{1}(Type\;of\;SLE)+\beta_{2}(Number\;of\;SLEs)+\beta_{3}X_{3}\ldots \beta_{n}X_{n}+e$$ (2)

$$\log(\frac{MMD}{1-MMD})=\beta_{0}+\beta_{1}(Environmetal\;SLE)+\beta_{2}(Economic\;SLE)+\beta_{3}(Family\;SLE)+\beta_{4}(Crime\;SLE)+\beta_{5}(Number\;of\;SLEs)+\beta_{6}X_{6}\ldots \beta_{n}X_{n}+e$$ (3)

To assess effect modification by level of social capital on the relationship between SLEs and MMD, we used logistic regression using the binary MMD variable and linear regression using a total woman’s score on the SRQ-20 as a continuous outcome. We also explored different cutoff values for CSC. We extended Model 3 to include an interaction term.

As the data for this study were generated by a cluster-based sampling strategy at the community level, robust standard errors were used to account for the inter-cluster correlation across observations. Data management and analysis was conducted using Stata version 14.0 [38] and graphics were produced using the R package *ggplot2* [39].

## Results

The final sample consists of 5,485 women (n = 1,703 in Ethiopia, n = 1,1792 in India, and n = 1,849 in Vietnam); 558 women were excluded because of missing data. Data are assumed to be missing at random as no patterning was found among excluded women. The percent of women missing data on specific variables ranged from 0% to 5.6% across countries.

Table 1 presents the distribution of MMD by country and individual characteristics. The proportion of women in the sample with suspected MMD was similar in Ethiopia (32.1%) and in India (29.7%), but much lower in Vietnam (21.0%). On average, women experienced 1.9 SLEs in Ethiopia (range: 0–10 SLEs), 1.08 SLEs in India (range 0–15 SLEs), and 0.6 SLEs in Vietnam (range 0–5 SLEs). Fig 1 highlights the considerable variation in symptomology of MMD in each country.

**Table 1:** Sample characteristics in Ethiopia, India, and Vietnam.

Sample Characteristics	Ethiopia % (n)	India % (n)	Vietnam % (n)
Number of Women	1703	1792	1849
% of Women with Maternal Mental Distress (n)	32.1 (547)	29.7 (531)	21.0 (388)
Mean number of Stressful Life Events (SD)	1.9 (1.7)	1.07 (1.6)	0.6 (0.8)
% of Households Headed by Mother (n)	8.5 (144)	0.73 (13)	58 (108)
Mother's Mean Age in Years (SD)	27.3 (6.2)	23.7 (4.3)	27.2 (5.8)
Marital Status			
% Married or Permanent Partner (n)	88.1 (1501)	99.6 (1784)	97.9 (1810)
% Divorced or Separated (n)	8.0 (137)	0.3 (6)	1.1 (20)
% Single (n)	3.8 (65)	0.1 (2)	1.0 (19)
Mean Cognitive Social Capital Score (SD)	3.5 (0.8)	3.4 (0.7)	3.6 (0.7)
Mother's Educational Level			
% Did not complete primary (n)	77.5 (1319)	59.7 (1069)	27.1 (501)
% Primary or above (n)	22.6 (384)	40.4 (723)	72.9 (1348)
Place of Residence			
% Urban (n)	35.5 (605)	25.3 (454)	19.9 (368)
% Rural (n)	64.7 (1098)	74.7 (1338)	80.1 (1481)
Mean Parity (SD)	3.6 (2.3)	2.0 (1.2)	1.9 (1.2)

Table 2 presents univariate and Table 3 presents multivariable adjusted logistic regression results obtained from Model 1. In Table 2, the number of SLEs and the strength of CSC is inversely associated with MMD in all three countries. In Table 3, the number of SLEs to which a woman was exposed remains significantly associated with MMD after adjusting for all variables in the final model. In all three countries, the results of the adjusted models suggest a positive relationship between the number of SLEs and the odds that a woman will suffer from MMD. For each additional SLE to which a woman was exposed, the odds of MMD increased by 1.3 (95% CI: 1.2, 1.4; p<0.001) in Ethiopia, 1.17 (1.1, 1.3; p<0.001) in India, and 2.0 (1.8, 2.3; p<0.001) in Vietnam. Additionally, the effect of CSC also remains significant in the multivariable adjusted models across all three countries: 0.6 (0.6, 0.7; p<0.001) in Ethiopia, 0.6 (0.5, 0.7; p<0.001) in India, and 0.7 (0.6, 1.9; p<0.001). Other variables potentially related to different dimensions of insecurity also remain significant in the multivariable adjusted model, with some heterogeneity across countries. In Ethiopia and Vietnam, women who are divorced, separated, single or widowed are significantly more likely to face MMD when compared to women who are married or cohabitating, while in India, women who are the head of household are more likely to experience MMDs than women who are not. Increasing wealth only appears to have a protective association against MMD in India, except for among the richest quintile in Vietnam. In India and Vietnam, having additional adult members of the household (aged 16 years and above) may have a protective effect given its significant inverse association with MMD.

**Table 2:** Odds ratios obtained from univariate logistic regression of common mental disorders on sociodemographic characteristics in Ethiopia, India and Vietnam.

	Ethiopia	India	Vietnam
Number of stressful life events in year before child’s birth	1.28 (1.22, 1.36)***	1.17 (1.11. 1.25)***	1.98 (1.75,2.25)***
Mother is head of household (ref: no)			
Yes	1.73 (1.22, 2.44)**	4.38 (1.34, 14.27)**	1.55 (1.02,2.34)*
Wealth Quintile (ref: poorest)			
Poorer	1.00 (0.72, 1.38)	1.24 (0.92,1.67)	0.83 (0.58, 1.18)
Middle	0.84 (0.60, 1.17)	0.71 (0.53,0.97)*	1.36 (0.97, 1.89)
Richer	1.16 (0.85, 1.59)	0.69 (0.51,0.93)*	0.76 (0.53, 1.08)
Richest	0.90 (0.65, 1.23)	0.27 (0.19,0.38)***	0.60 (0.42, 0.88)**
Cognitive Social Capital Score	0.64 (0.56, 0.72)***	0.67 (0.57, 0.78)***	0.65 (0.57, 0.75)***
Mother's Educational Level (ref: none)			
Completed Primary	0.84 (0.11, 1.32)	0.50 (0.40,0.61)***	0.77 (0.61, 0.97)*
Member of Ethnic Majority (ref: no)	1.00 (0.77, 1.28)	0.77 (0.61,0.99)*	1.04 (0.79, 1.38)
Member of Religious Majority (ref: no)	1.15 (0.92, 1.43)	0.98 (0.73,1.32)	0.93 (0.71, 1.20)
Place of Residence (ref: urban)	0.84 (0.68, 1.04)	1.94 (1.52,2.48)***	0.93 (0.71, 1.20)
Mother's Age (years)	1.02 (1.0, 1.04)***	1.02 (1.00, 2.05)*	1.01 (1.00, 1.03)
Time lived in Community (years)	1.01 (1.00, 1.50)	1.02 (1.00,1.03)**	1.01 (1.00, 1.03)*
Marital/Cohabitation Status (ref: Married)			
Married–Living Apart	1.43 (0.64, 3.17)	0.56 (0.24,1.31)	0.78 (0.16,3.69)
Divorced/Separated/Single /Widowed	1.76 (1.31, 2.38)***	3.19 (0.76,13.42)	4.04 (2.12,7.67)***
Number of Children Under 5 years in Household	1.00 (0.83, 1.20)	1.23 (1.01,1.49)*	1.98 (1.75,2.25)
Number of Children between the ages of 5–15 years in Household	1.12 (1.04, 1.20)**	1.18 (1.07,1.30)***	1.00 (0.99,1.01)
Number of Individuals aged 16+ years in the Household	1.10 (1.01, 1.21)*	0.90 (0.85,0.96)***	0.85 (0.78,0.93)***

**Table 3:** Odds ratios obtained from multivariable logistic regression of common mental disorders on sociodemographic characteristics in Ethiopia, India and Vietnam^1^.

	Ethiopia	India	Vietnam
Number of stressful life events in year before birth	1.30 (1.22, 1.39)***	1.09 (1.02, 1.17)**	1.90 (1.66, 2.17)***
Mother is head of household (ref: no)	--	5.07 (1.28, 21.11)*	
Wealth Quintile (ref: poorest)			
Poorer	1.00 (0.71, 1.40)	1.24 (0.92, 1.68)	0.75 (0.50, 1.10)
Middle	0.74 (0.52, 1.05)	0.73 (0.53, 0.99)*	1.21 (0.81, 1.78)
Richer	0.95 (0.63, 1.43)	0.70 (0.51, 0.96)*	0.73 (0.49, 1.09)
Richest	0.78 (0.49, 1.24)	0.26 (0.18, 0.38)***	0.67 (0.43, 1.05)
Cognitive Social Capital Score	0.63 (0.55, 0.72)***	0.61 (0.52, 0.72)***	0.66 (0.58, 1.92)***
Member of Ethnic Majority (ref: no)	--	--	1.29 (0.85, 1.95)
Member of Religious Majority (ref: no)	--	0.78 (0.56,1.07)	--
Place of Residence (ref: urban)	0.68 (0.47, 0.97)*	--	--
Mother's Age (years)	1.00 (0.97, 1.02)	1.01 (0.98, 1.04)	1.01 (0.98, 1.03)
Marital/Cohabitation Status (ref: Married)			
Married–Living Apart	1.33 (0.56, 3.17)	--	1.66 (0.33, 8.52)
Divorced/Separated/Single	1.80 (1.29, 2.52)***	--	3.51 (1.75, 7.05)***
Number of Children Under 5 Years in Household	--	1.06 (0.89,1.37)	--
Number of Children Aged 5–15 Years in Household	--	--	0.90 (0.76, 1.05)
Number of Individuals Aged 16+ Years in the Household	--	0.92 (0.86, 0.97)**	0.90 (0.82, 0.99)*
Parity	1.10 (1.02, 1.18)**	1.05 (0.95, 1.17)	--

^1^ Covariates included in each multivariable model determined through backward elimination

**Fig 2:**
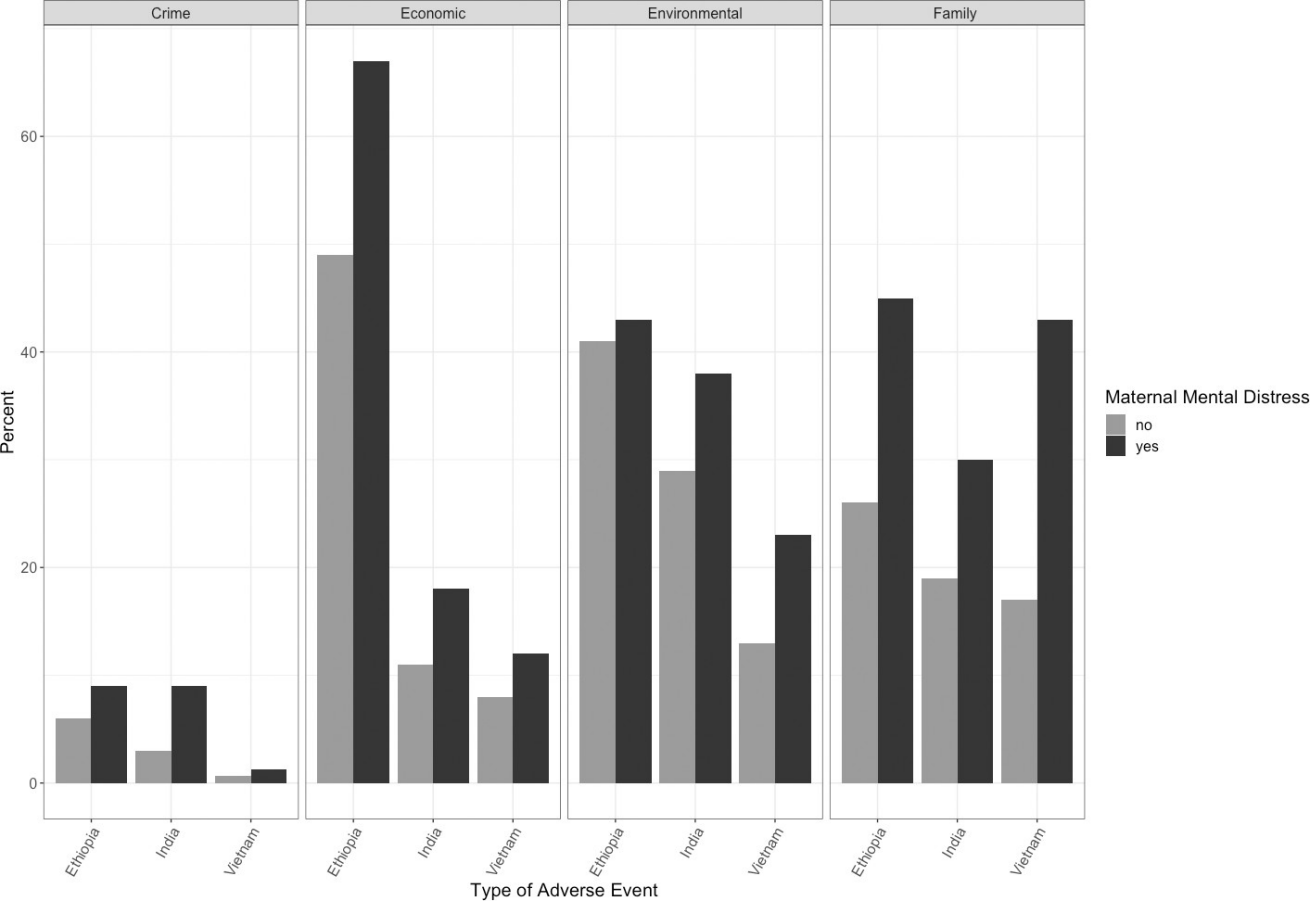
Distribution of the type of Stressful Life Event (SLE) experienced by women in the 12 months prior to giving birth in Ethiopia, India, and Vietnam according by MMD status.

Fig 2 presents the distribution of the type of SLE according to country and MMD status. The percentage of women experiencing each type of SLE is highest in Ethiopia, followed by India and Vietnam for all but family SLEs. In all countries, exposure to SLEs related to crime is much less common than the other types of SLEs.

The results presented in Table 4 highlight the association between different types of SLE and MMD obtained from Models 2 and 3. In unadjusted models, nearly all types of SLEs were significantly associated with MMD (except for environmental SLEs in Ethiopia and crime SLEs in Vietnam). Results from Model 3 suggest that exposure to family SLEs is significantly associated with MMD in all three countries with odds ratios of 1.8 (95% CI: 1.3, 2.4; p<0.001), 1.62 (95% CI: 1.1, 2.3; p<0.01 in India), 1.9 (95% CI: 1.3, 2.9; p<0.01), respectively. In Ethiopia and India, economic SLEs were also significantly associated with MMD after adjustment (Ethiopia OR: 1.7; 95% CI: 1.1, 2.5; p<0.01 and India OR: 1.4; 95% CI: 1.0, 2.1; p<0.05), while in India, crime SLEs (OR: 1.9; 95% CI: 1.3, 2.9; p<0.01) were associated with MMD.

**Table 4:** Odds ratios obtained from unadjusted and multivariable logistic regression analysis decomposing the association between adverse life events and presence of common mental disorder by type of Stressful Life Event (SLE) experienced in the 12 months prior to giving birth in Ethiopia, India, and Vietnam.

	Unadjusted OR (95% CI)	Model 2:	Model 3:
		Multivariable-adjusted OR^1^ (95% CI)	Multivariable-adjusted OR^2^ (95% CI)
**Ethiopia**	** **		
Environmental SLE	1.04 (0.85, 1.28)	0.56 (0.41, 0.77)***	0.74 (0.52, 1.05)
Economic SLE	1.99 (1.62, 2.46)***	1.39 (0.95, 2.04)	1.68 (1.12, 2.52)**
Family SLE	2.44 (1.97, 3.02)***	1.78 (1.36, 2.33)***	1.76 (1.30, 2.38)***
Crime SLE	1.68 (1.14, 2.50)**	1.36 (0.89, 2.07)	1.36 (0.88, 2.09)
**India**	** **		
Environmental SLE	1.39 (1.13, 1.71)**	0.80 (0.57, 1.11)	1.15 (0.79, 1.69)
Economic SLE	1.73 (1.31, 2.29)**	1.24 (0.90, 1.71)	1.44 (1.01, 2.05)*
Family SLE	1.77 (1.41, 2.22)***	1.44 (1.03, 2.00)*	1.62 (1.12, 2.33)**
Crime SLE	2.39 (1.58, 3.61)***	1.85 (1.13, 3.02)*	2.15 (1.27, 3.63)**
**Vietnam**	** **		
Environmental SLE	1.92 (1.45, 2.54)***	0.68 (0.47, 0.98)*	0.95 (0.60, 1.51)
Economic SLE	1.53 (1.06, 2.21)*	2.03 (1.76, 2.35)***	0.79 (0.49, 1.28)
Family SLE	3.69 (2.89, 4.07)***	2.09 (1.53, 2.87)***	1.93 (1.27, 2.92)**
Crime SLE	1.37 (0.43, 4.33)	0.52 (0.15, 1.77)	0.62 (0.18, 2.20)

* p<0.05

** p<0.01

*** p<0.001

^1^ Models each type of adverse events in a separate model. Ethiopia: Model adjusts for number of adverse events, place of residence, wealth quintile, cognitive social capital, age, partner living arrangement, and parity. India: Model adjusts for number of adverse events, female-headed household, cognitive social capital, wealth quintile, maternal age, religion, number of children under the age of 5 years in house, number of adults over 16 years in house, and parity. Vietnam: Model adjusts for number of adverse events, cognitive social capital, ethnic majority, wealth quintile, age, number of children under 5 years in house, and partner living arrangement.

^2^ Adjusted for all variables in Model 2 and also for the co-occurrence of other types of SLEs

**Fig 3:**
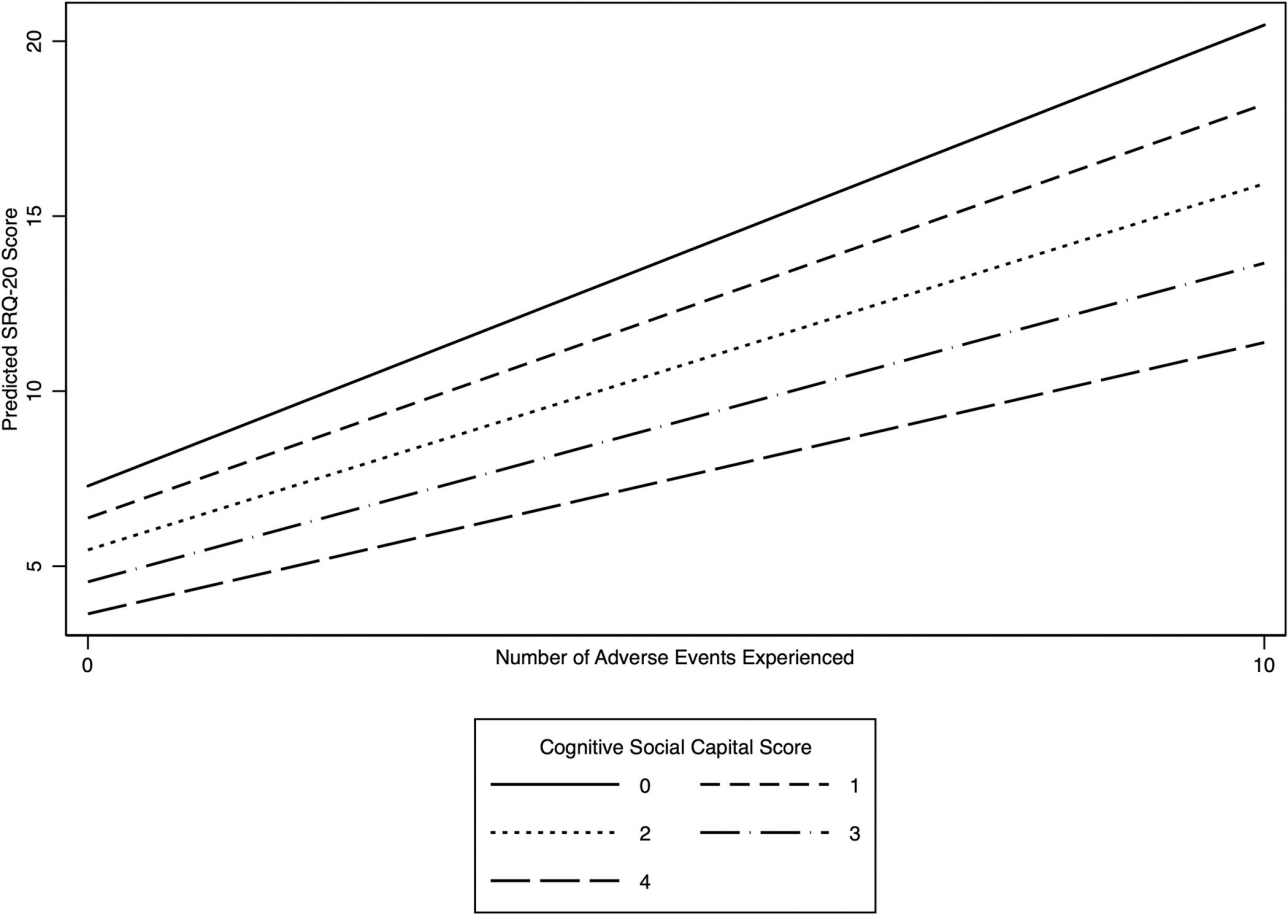
Adjusted predicted SRQ-20 score by number of stressful life events experienced for different levels of cognitive social capital in Ethiopia.

We find some variation in effect modification across countries after adding interaction terms between the number of SLEs and CSC to Model 3. In examining SRQ-20 score as a continuous variable, in Ethiopia, we find that as social capital increases, the lesser the effect SLEs have on maternal mental health holding other variables equal (p<0.05). The results suggests that for an average women with a social capital score of 0, her SRQ-20 score would increase by 9.7 points (95% CI: 8.6, 10.7) for each additional SLE she experienced in the one year prior to giving birth, while the SRQ-20 score for an average woman with a social capital score of 4 would only increase by 5.1 (95% CI: 4.9, 5.3) for each additional SLE she experienced. Fig 3 illustrates the interaction effect observed in Ethiopia. In India and Vietnam, the interaction term between the continuous specification of variables relating to the number of SLEs and social capital score on SRQ-20 score was not significant. However, we explored the possibility for interaction based on categorical versions of the variables based on their distributions. In India, we categorized social capital score into high (3–4 points) versus low (2 points or lower)[40] and whether a woman had a SLE or not. Exploring the interaction this way yielded a significant result (p<0.01) whereby an average women with high social capital who had experienced at least one SLE would have an SRQ-20 score 2.14 points lower (95% CI: -3.8, 0.5) than an average women with low social capital who also had experienced at least one SLE. In Vietnam, we only find a significant interaction between SLEs and CSC among women with more than three SLEs. Among such women, women with a high social capital score on average experience an SRQ-20 score 3.0 points lower (95% CI: -5.8, 0.2) than women with a low social capital score.

## Discussion

This study highlights several important factors related to adversity and MMD in LMICs. First, the results suggest that in all three study countries, cumulative exposure to SLEs during the 12 months prior to giving birth was associated with increased mental distress. Second, the association between MMD and the types of adversity experienced varied by country. Finally, we find that CSC may serve as a buffer between exposure to adversity and MMD.

Our results suggest that the effect of SLEs on mental distress appears to be cumulative in Ethiopia, India, and Vietnam. Women in households that experience more SLEs within a short period of time may find it more difficult to recover and cope with the changes to household well-being, and coping strategies may further exacerbate maternal mental health, such as eating less and reducing household assets [41]. Surprisingly, our results did not find a clear relationship between wealth and MMD, except for in India. In many countries, women suffering from poverty or financial hardship have been found to suffer from higher mental distress than wealthier or more financially stable women [4, 16, 42–50]. Chronic poverty, material deprivation, and low socioeconomic status is consistently associated poor mental health among mothers [16, 44, 51–56]. Our results may be partially attributable to the sampling approach used in YL whereby participants were oversampled from the poorest and most disadvantaged communities [27]. As such, the wealth quintiles were constructed within a poor sample and may not reflect the full range of wealth that exists within a country. It is possible that the wealthiest households included in YL are still not wealthy enough for the relative increases in wealth to have a protective effect on maternal mental health.

The types of SLEs associated with MMD may be important for intervention design. The importance of family-related SLEs in all three countries indicates that household disruption may be particularly devastating to mothers in the perinatal period. A study using YL data found that paternal death was often considered to be the most severe event with regards to a household’s well-being [57]. Awareness-raising within communities, or screening during provider contacts with perinatal women, could improve the identification of women coping with adverse family events. Interventions could be tested that aim to improve the social resources available to women suffering from family-related SLEs in order to reduce their risk for adverse mental health outcomes [58]. Economic SLEs emerged as important in India and Ethiopia. In Ethiopia, economic SLEs were the most common type of SLE experienced, and were also more common than in the other study countries. As we only have data available on a limited list of economic SLEs in this study, it is possible that the economic hardships most pertinent to the other countries in the study were not included, which may represent a direction for future research. In India, being exposed to a crime-related SLEs was associated with MMD, despite the relatively low prevalence of such SLEs. A study in South Africa suggests that being exposed to crime may relate to postnatal depression by eroding trust in the community environment [59] and as such, may be particularly relevant to interventions focused on CSC.

Finally, this study suggests that CSC may serve as a buffer between SLEs and maternal mental health. While this is the only study that we know of to demonstrate this effect, there is some heterogeneity in the effect observed across countries. In all three countries, higher CSC was associated with reduced MMD. We found the most robust evidence for an interaction between CSC and SLEs on maternal mental health in Ethiopia. The results in India and Ethiopia may suggest a threshold of SLEs (more than one and three, respectively) at which CSC begins to operate to reduce a woman’s SRQ-20 score. These differing results could be the result of several factors. The economic literature on SLEs differentiates between 1) common SLEs, defined as those that affect everyone within a community, and 2) individual SLEs, defined as those that only affect an individual. A study from a later wave of YL found that when SLEs occurred in Ethiopia, they were more likely to collectively affect members of the same community than in the other study countries, where SLEs are more often experienced individually [57]. In Ethiopia, it is common for post-partum women to receive additional support from their families, relatives, and neighbors. Of note, the multivariable regression results in Vietnam and India both suggest that having additional adult members in the household (aged 16 years or more) is protective of maternal mental health, whereas in Ethiopia, there is no evidence of an effect. Perhaps the variability in this relationship across countries is because women in Ethiopia rely to a greater extent on their community than on their individual household for support as compared to women in India and Vietnam. Interestingly, previous studies suggest some heterogeneity across countries in relation to whom women draw on for support. Studies in high income countries have found that mothers rely on coping resources from within their close interpersonal relationships [13], while a study conducted in Malawi found that women believed that a lack of financial or emotional support from friends and family members during the perinatal period was a risk factor for mental distress [60].

Another explanation of the heterogeneity in effect modification could relate to the measure of social capital itself. Cognitive social capital focuses on an individual’s perception of their communities, and as such, it may be particularly well-suited to help women cope with the shared nature of the SLEs experienced in Ethiopia. Arguably, CSC deals with fundamental emotional perceptions of one’s community, and may not vary culturally to the same degree as other measures of social capital [40], which focus on elements of individual behavior, such as participation in community groups [61]. Future research could examine the influence of other types of social capital or social support in these contexts. Interventions to improve maternal social capital may help to improve the mental health of women suffering from SLEs. Identifying intervention to change the way a woman feels about her community is somewhat difficult; however, supporting women to forge new supportive networks by increasing her structural social capital through community group membership may reduce her sense of social exclusion and therefore change her perceptions of her community [40]; however, there is still limited evidence of the effectiveness of such interventions [62]. Interestingly, another study using YL data found that increased group membership among women in Ethiopia may actually have a negative influence on maternal mental health [63]. It may be the case that among these women, the added burden of group participation may aggravate existing mental distress [64]. More research in this area would help to clarify these pathways across countries and clarify intervention approaches.

This study should be interpreted in light of several limitations. We cannot assess causation given that this is an observational study using secondary data. Women were only asked to report SLEs that they believed to be important, meaning that their response may be dependent upon cultural and social norms related how they interpret the severity of the event. Women suffering from MMD may be more likely to perceive a past life event as important. Additionally, the timing of survey administration makes it impossible to determine the time of onset of the distress as no data are available on a woman’s mental health prior to their postpartum assessment or during the first 6 months post-partum, a time during which depression is most common. Without data on onset, we cannot specifically assess perinatal mental health disorders. Additionally, we were unable to find a precise definition to describe our outcome of interest, as our exposure occurred one year before childbirth, and the study period for assessing the outcome corresponds to six to 18 months post-partum. We decided to describe our outcome as MMD as the term maternal is broadly encompassing; however, maternal usually refers to the time between the onset of pregnancy and one year postpartum. Further, not having data on onset could make the results susceptible to reverse causation, as some SLEs could be caused by MMD (such as divorce). Additionally, we recognize that there is some overlap in the categorization of SLEs. For example, a crime related SLE that results in the loss of livestock may result in economic turmoil for a family. However, we focus on the origin of the SLE, not the mechanism for action in determining its categorization. The YL dataset should not be interpreted as being nationally representative given YL’s poor-biased sampling approach [27] Lastly, the index child in the survey must have been alive at the time of enrollment, thus the associations observed in this study may be weaker than would be otherwise observed if women who have a pregnancy that does not result in a live birth or women who experience the death of a child are more likely to experience MMD [65].

In conclusion, this study suggests a complex relationship between SLEs, CSC and MMD in three diverse LMICs. Longitudinal studies that are not limited by the cross-sectional design of this study are needed to better understand the temporal relationship between the variables studied. The findings of this study may help programs in the identification of the most vulnerable women, and point towards the potential for interventions designed to improve perinatal mental health outcomes by increasing social capital, especially as our results highlight the possible role that social capital may play in buffering the effect of SLEs on MMD. Interventions designed to reduce the risk of MMD may be most important in contexts where women face multiple adversities combined with layered disadvantage and limited social capital [6].
